# Improving patient flow in acute psychiatric wards: enhanced bed management and trusted assessment

**DOI:** 10.1192/bjb.2020.12

**Published:** 2020-08

**Authors:** Douglas Turkington, Steve Moorhead, Gordon D. Turkington, Carla King, Leigh Bell, Denise Pickersgill

**Affiliations:** 1 Monkwearmouth Hospital, Cumbria, Northumberland, Tyne and Wear NHS Foundation Trust, Sunderland, UK

**Keywords:** In-patient treatment, cost-effectiveness, psychiatric nursing, outcome studies, psychosocial interventions

## Abstract

**Aims and method:**

In three localities in a mental health trust in England, an enhanced bed management team was established to improve patient flow and reduce out-of-area placements. Trusted assessments were provided to support risk management and conflict resolution. Two measures of flow were compared before and after the team was established.

**Results:**

The trusted assessment recommendation was for discharge in 70% of cases. The number of out-of-area placements was significantly reduced (*P* < 0.05), saving £616 876 over a 12-month period. Patient flow was significantly improved in one of the three localities as measured by patients/bed/6-month period (*P* < 0.05). In one of the other localities increased use of trusted assessment input and reduced numbers of patients being transferred in are recommended to improve flow.

**Clinical implications:**

Mental health trusts should consider the establishment of an enhanced bed management team, including trusted assessment, as a safe and cost-effective approach to improving patient flow and reducing the need for out-of-area placement.

NHS England's Five Year Forward View for Mental Health^1^ mandated that access should be increased to high-quality community care, avoidable admissions should be prevented and treatment delivered as close to home as possible (recommendation 22). Out-of-area placements were to be completely eliminated by the financial year 2020–2021. ‘Financial incentives and levers’ were to be deployed to deliver on the key outcomes anticipated in recommendation 22. Consequently, mental health trusts working under contracts where these levers were being deployed had been losing substantial amounts of non-returnable funding through the use of out-of-area placements at a cost of £608/day. It is known that patient outcomes when admitted out of area are significantly worse than for those admitted locally.^2^ The Five Year Forward plan, however, set targets without acknowledging that many mental health trusts were already ‘under-bedded’ in relation to their population size and level of social adversity and under-resourced in terms of community provision. Also, critical indicators for admission/discharge and estimated discharge dates were being underutilised by crisis teams and ward staff, leading to unnecessarily prolonged in-patient stays.^3^ Meanwhile, since the plan was written and distributed, a number of factors have come together to increase the pressure on scarce in-patient bed resources.^4^ In particular, the percentage of patients admitted under the Mental Health Act 1983 has increased, as has length of stay. Intellectual disability (‘learning disability’) beds were being reduced for both the forensic and working-age adult populations. One possible explanation was put forward that clients are declining informal admissions as the acute wards are perceived as stressful and end up eventually being admitted for a longer period under the Mental Health Act.^5^ This is paralleled by nursing staff opinion that acute in-patient wards are now best viewed as ‘mini-PICUs’. Reduction in the use of the Mental Health Act 1983 is an objective of the Independent Review of the Act (which was completed in December 2018 and for which a white paper is proposed in the next session of parliament), and this could potentially improve flow through acute admission beds. Over the past 5 years there has been a 50% increase in accident and emergency (A&E) department attendances for mental health problems.^6^ This has led to increased pressure on crisis and liaison specialist services and, at times, perhaps unnecessary admission under the Mental Health Act. Presentations with intoxication from alcohol and other substances have been rising. We have also seen a surge in the use of ‘gate sections’ from Her Majesty's Prisons. This occurs when a prisoner has been mentally ill, was not transferred to prison under section 38 but is detained under the Act ‘at the gate’ at the time of their release. People whose primary diagnosis is a personality disorder are frequently kept too long under assessment in in-patient units, where risk may increase rather than decrease. Their difficulties have often arisen from prolonged or repeated traumatising experiences in childhood or adolescence, and they may be better supported with effective community psychological management programmes,^7^ avoiding off-licence use of psychopharmacology and potentially further traumatising coercive in-patient treatment under the Mental Health Act.^8^ Any such admissions should be brief and require a team or pathway-based attitude supporting positive risk management. Risk averse practice has been rising owing to the negative consequences ensuing if the discharge and community plan are not effective in preventing a serious adverse outcome. All of these factors have combined to make the current in-patient experience of care potentially a stressful one for patients and staff alike.

There is no doubt that the spiralling development of specialist teams (while a good thing for their target populations) has significantly weakened the generic community mental health teams. Waiting lists for key worker allocation are lengthening and, at times, individuals are discharged too soon, removing ongoing support with well-being activities (including medication concordance support). Another factor that has driven admissions up is that nobody ‘owns the flow’. Sector and integrated psychiatric services would facilitate a discharge if an admission was needed. However, ‘split’ in-patient and out-patient consultant psychiatrist posts have contributed to impairing pathway ownership of the flow of patients into and out of acute units as well as causing patient dissatisfaction with perceived lack of continuity of care.^9^ As a result of this accumulation of factors, many trusts across the UK have found themselves in great need of a solution to the problem of lack of available admission beds that arises as they do not have ‘the right patient at the right place at the right time’ on their care pathway. Cumbria, Northumberland, Tyne and Wear NHS Foundation Trust (NTW), which covers a population of approximately 1.5 million, decided that effective and prompt discharge to the correct treatment pathway needed to be facilitated and the trust board mandated a director to take responsibility for this crucial area. The enhanced bed management (EBM) service was created as one of a number of interlinking strands to deal with rising demand for in-patient admission. The other strands included the establishment of a personality disorder hub, improving crisis assessment and home treatment teams and improving rehabilitation flow. This paper reports early findings from evaluation of our EBM service.

## Method

In response to the above pressures, NTW decided to implement the EBM approach across all acute psychiatric wards in three localities. The EBM team included three discharge facilitators and five bed managers. There were also one nurse manager, one part-time administrator, one part-time social worker, one research assistant and two part-time consultant psychiatrists (both 0.4 full-time equivalent). A system of linked detailed electronic bed boards was introduced in all acute wards trust-wide to regularly update critical indicators, estimated discharge date, mental health cluster and Mental Health Act status. Discharge facilitators attended all multidisciplinary team (MDT) meetings and clinical bed managers were available for consultation on a locality basis. ‘Trusted assessments’ were made available on request by the MDT with the agreement of the responsible clinician. The purpose of the trusted assessment was to complete a full review of the history, interview the patient and all pertinent staff and give an independent and comprehensive opinion to all parties on diagnosis, treatment and management. Trusted assessments began in January 2018, continued throughout that year and are ongoing. The impact of EBM on out-of-area placements (adult acute) was calculated by comparing a proxy of flow (number of out-of-area placements/month) between the calendar years of 2017 and 2018. The impact on flow as measured by patients/bed was compared between the first 6 months and second 6 months of 2018 (the second reflecting the period when the full team was operational). Unpaired *t*-tests were used to compare the periods in question.^10^ All other statistical analyses were performed using IBM SPSS Statistics (for Windows), release 24.0.0.2. Further exploration to understand the impact of internal transfers examined length of stay data for all patients discharged from adult acute wards in the trust during the financial year 2018–2019. Outcomes and recommendations from the first 50 trusted assessments were determined by case note review in March 2019.

Ethical approval was not sought as this project was a service evaluation and there was no randomisation and no treatment being tested.

## Results

### Out-of-area placements

The number of out-of-area placements was reduced by over 60%: in 2017 the monthly mean was 5.25 (65 placed in the year); in 2018 the mean was 2.4 (29 placed in the year) (*P* < 0.05). This equated to a saving of £616 876 in otherwise lost revenue (the figure given for savings does not include the cost of the EBM team).

### Patient flow

Flow (patients/bed/6-month period) showed a significant improvement in one locality (*P* < 0.05) in the period of full operation of EBM, compared with the preceding 6 months: 4.83–5.5 (167 admissions rising to 246, with 56 transfers reducing to 52 over that period). In the other two localities one already had acceptable levels of flow and these did not change significantly (5.2–5.36; 253 admissions rising to 260 and 56 transfers reducing to 35). In the other locality flow remained lower, at 4.6–4.65 (284 admissions reducing to 253 and 46 transfers increasing to 71). The three localities had 57, 70 and 54 acute beds respectively. Patient flow is locality specific and deemed acceptable in two NTW localities, because if all three localities were hitting the same flow targets of 5.2–5.5 patients/bed/6-month period then there would have been no out-of-area placements and only infrequent admissions into leave beds.

### Trusted assessment uptake

To examine the potential impact of trusted assessment uptake on locality patient flow, a *post hoc* correlation between number of trusted assessments provided and proportionate increase in flow was calculated. In locality 1, where 9 trusted assessments were requested, the proportionate change in flow was −0.13. The flow figures for locality 2 were 0.02 (with 14 trusted assessments) and for locality 3 they were 0.13 (with 19 trusted assessments). A correlation between proportionate change in patient flow and number of trusted assessment requests was significant, with a two-tailed Spearman's rho of 1.0; *P* < 0.001.

### Length of stay

A more detailed exploration of the factors affecting flow data was undertaken. Initial flow data indicated that flow was noticeably low on one ward in particular. The consultant body suggested that looking after relatively more intra-trust, cross-locality ‘transfers in’, who would, by implication, be more ill, might explain the lower flow. Examination of data for 1 year of patients who had been discharged showed that intra-trust, inter-locality transfers indeed stayed significantly longer than those who were admitted and discharged from the same ward (mean stay 70 days compared with 32 days; *P* < 0.001). If short-stay patients (in for less than 20 days) are removed from this analysis, a statistically significant difference remained (83 *v*. 59 days; *P* < 0.001). Overall then, intra-trust transfers stay significantly longer than those remaining on the ward on which they land and this ward had a much greater proportion of transfers in. However, both male wards in this locality had, proportionately, a considerably greater number of transfers in, contributing to lower flow data for the whole locality. Clarity, then, about the greater numbers of transfers in and their associated length of stay initially suggested an explanation for this low flow (many more transfers in, who stay longer). However, further analysis of the pathway indicated that these patients had remained on initial wards before the transfer for a mean of 18 days. If the same group of short-stay patients are again removed, the mean rises to 21 days, corresponding almost exactly to the difference in mean of total length of stay between the groups of those transferred and those remaining (24 days) once the short-stay group was removed from the data. Thus, the length of stay on the wards on which the patients land after the initial stay was examined. This showed that male transferred-in patients in this locality as a whole stayed significantly longer after their arrival on the destination ward than male transfers in in the other localities (means: 38, 46 and 71 days; one-way ANOVA, d.f. = 2; *F* = 4.6; *P* = 0.01) indicating a difficulty with the male pathway as a whole in this locality rather than just one ward.

### Trusted assessment recommendations

Examination of ‘the first 50’ outcomes showed that the trusted assessments recommended discharge for 35 (70%) of patients they were asked to assess and, of these, 19 were discharged within 2 weeks; 25 of the 35 were discharged within 4 weeks. There were no untoward incidents in the follow-up period after discharge (which was obviously different for each patient, depending on the timing of trusted assessment provision): the mean was 149 days (range: 89–355). This amounted to 3730 people-days among 25 people. Thirteen of this 25 experienced a readmission (eight had one readmission, four had two and one had three readmissions) for a median of 9 days in total. Considering the impact of the trusted assessment on overall care, this 25 had experienced a mean of 7321 days as in-patients since their very first admission and 21 863 days living in the community (ratio 0.33). In the intervening 3730 people-days, these 25 patients experienced a mean of 35.4 days as in-patients and 251.5 in the community, a ratio of 0.14.

## Discussion

These results show that, by investing in an enhanced bed management (EBM) service, improvement in quality of care and substantial financial savings can be achieved by preventing unnecessarily long hospital stays.^11^

### Trusted assessments

Although the clinical bed managers, discharge facilitators, EBM social worker and research assistant were broadly welcomed by in-patient teams, the role of the trusted assessment was viewed initially with some suspicion, as the exact nature of the role was not understood for some time. The initiation of the concept of trusted assessment required the agreement of the responsible clinician. There was not a uniform uptake across the three localities. This might indicate ambivalence on the part of the MDT or the responsible clinician. We are not aware of any vetoing of a trusted assessment by the responsible clinician when it was requested by the MDT. In a parallel project to build consensus there was 89% agreement with the following statement among a multiprofessional consultant staff group: ‘Given consensus that the needs of current in-patients should be balanced with the needs of those waiting admission, a trusted assessment is helpful in contributing a view that explicitly takes account of the wider needs of the system and when such needs are incorporated into the trusted assessment thinking these should be explicitly articulated in the report’. A trusted assessment is only undertaken at the request of the MDT and with the full consent of the responsible clinician. Trusted assessments were able to support the MDT in relation to difficult discharge situations.

The trusted assessments recommended prompt discharge in 70% of cases and were able to support MDTs in terms of mediation between different views and positive risk management to achieve prompt discharge.

Specific locality-based analysis of patient flow highlighted difficulties that required detailed analysis of data on length of stay to fully understand local problems, ensuring that possible solutions could be developed. Rather than showing that transfers in alone explained the problem on one ward, this analysis revealed that there were whole-locality pathway problems. A number of patients were waiting for a rehabilitation bed and there were fewer discharge options in terms of supported accommodation in that locality. Further, the locality in question did not make use of trusted assessments, whereas the locality that optimised flow was a heavy user of trusted assessments.

Trusted assessment has been operating in acute hospitals for some time but with a slightly different role, where a number of different providers agree that the trusted assessment will decide on the most appropriate discharge package once that discharge has been decided on.^12^ Our model of trusted assessment, within mental healthcare, is that the various teams within the trust agree to clinical mediation, positive risk management or other care strategy with the contribution of an experienced clinician working within EBM. This team can be consulted in relation to EBM establishment and working practice, and the multimodal linked bed boards viewed. If the important targets of the Five Year Forward plan are to be achieved within a system of suboptimal bed provision, our findings show initial support for the contention that EBM, incorporating trusted assessments, is a safe and viable option.

### Study limitations

This is the first publication of the impact of such a service. Different comparison periods were used for out-of-area placements and flow because trusted assessments began in January 2018 but the full EBM team was not functioning until mid-2018. It is likely that the impact on out-of-area placements when next measured will be further enhanced. A further limitation of this report is that the case note review of the impact of the first 50 trusted assessments was done by a non-masked team member.

### Implications for other mental health trusts

This service development took place in a mental health trust that already had an ‘outstanding rating’ from the Care Quality Commission. In-patient beds, rehabilitation beds, community and other resources were all close to the median in the NHS benchmarking document.^13^ The Five Year Forward plan, however, set targets without acknowledging that many mental health trusts were already ‘under-bedded’ in relation to their population size and level of social adversity and under-resourced in terms of community provision. It is certainly the case that there are mental health trusts where much higher numbers of out-of-area acute beds are chronically in use. If the important targets of the Five Year Forward plan are to be achieved within a system of suboptimal bed provision, our findings show initial support for the contention that EBM, incorporating trusted assessments, is a safe and viable option. The generalisation of these findings to other trusts and settings will depend on an adequate number of acute psychiatric beds being funded and a number of other locality-specific factors. These include the level of social deprivation and adequate funding of crisis/home treatment teams and other community mental health provision. Mental health trusts might consider appointing a senior clinician or director with responsibility for pathway synchronisation and ownership of patient flow. This model may also prove beneficial for older adult and rehabilitation services.

## About the authors

**Douglas Turkington**, MD, FRCPsych, is a Consultant Psychiatrist at Monkwearmouth Hospital, Cumbria, Northumberland, Tyne and Wear (NTW) NHS Foundation Trust, Sunderland, UK. **Steve Moorhead**, MRCPsych, is a Consultant Psychiatrist at Monkwearmouth Hospital, NTW NHS Foundation Trust, Sunderland, UK. **Gordon D. Turkington**, BSc, is a Higher Research Assistant at Monkwearmouth Hospital, NTW NHS Foundation Trust, Sunderland, UK. **Carla King**, BSc, is a Team Manager at Monkwearmouth Hospital, NTW NHS Foundation Trust, Sunderland, UK. **Leigh Bell**, BN, is a Bed Manager at Monkwearmouth Hospital, NTW NHS Foundation Trust, Sunderland, UK. **Denise Pickersgill**, BA, is Associate Director of Monkwearmouth Hospital, NTW NHS Foundation Trust, Sunderland, UK.
